# An 8-year point-prevalence surveillance of healthcare-associated infections and antimicrobial use in a tertiary care teaching hospital in China

**DOI:** 10.1017/S0950268818002856

**Published:** 2018-10-25

**Authors:** Yi-Le Wu, Xi-Yao Yang, Meng-Shu Pan, Ruo-Jie Li, Xiao-Qian Hu, Jing-Jing Zhang, Li-Qi Yang

**Affiliations:** Department of Hospital Infection Prevention and Control, The Second Affiliated Hospital of Anhui Medical University, Hefei 230601, Anhui, China

## Abstract

Healthcare-associated infections (HAIs) are a major worldwide public-health problem, but less data are available on the long-term trends of HAIs and antimicrobial use in Eastern China. This study describes the prevalence and long-term trends of HAIs and antimicrobial use in a tertiary care teaching hospital in Hefei, Anhui, China from 2010 to 2017 based on annual point-prevalence surveys. A total of 12 505 inpatients were included; 600 HAIs were recorded in 533 patients, with an overall prevalence of 4.26% and a frequency of 4.80%. No evidence was found for an increasing or decreasing trend in prevalence of HAI over 8 years (trend *χ*^2^ = 2.15, *P* = 0.143). However, significant differences in prevalence of HAI were evident between the surveys (*χ*^2^ = 21.14, *P* < 0.001). The intensive care unit had the highest frequency of HAIs (24.36%) and respiratory tract infections accounted for 62.50% of all cases; *Escherichia coli* was the most common pathogen (16.67%). A 44.13% prevalence of antimicrobial use with a gradually decreasing trend over time was recorded. More attention should be paid to potential high-risk clinical departments and HAI types with further enhancement of rational antimicrobial use.

## Introduction

Healthcare-associated infections (HAIs) have become a major public-health problem around the world, being associated with increased morbidity, hospital stay, long-term disability, antimicrobial resistance and healthcare costs [1, 2]. Prevalence surveys are important methods for evaluating infection control efforts and have been widely used to determine the range of HAIs and antimicrobial use in hospitals. For example, a recent multistate point-prevalence survey in the USA reported a rate of 4.0% HAIs among inpatients in acute care hospitals [3], and rates of 4%–9% have been recorded in similar European surveys [4–7]. Similarly in Southeast Asia, a pooled study reported a 9.0% overall prevalence of HAI [8], and in China, a multi-centre survey found a 3.7% prevalence of HAI [9].

Inappropriate use of antimicrobials has often been noted to be an important contributing factor for the increase of HAIs, particularly those associated with resistant strains caused by overuse of antimicrobial drugs [10]. Surveillance data published by the European Centre for Disease Prevention and Control (ECDC) underline the fact that therapeutic options are becoming increasingly limited in several countries with high levels of multidrug resistance [11]. It is therefore important to understand the status and reasons of antimicrobial use for accurate intervention.

Periodical hospital-wide point-prevalence surveys have been shown to be effective to gather data on the extent and trends of HAIs and antimicrobial use, which can help policy makers and hospital infection control personnel to establish appropriate targets and prevention strategies [12, 13]. Such surveys are performed annually in our hospital, to estimate the overall prevalence and characteristics of HAIs and antimicrobial use. There are less data concerning long-term trends of HAIs and antimicrobial usage in Eastern China, and these issues are addressed in the current study.

## Methods

### Study design and setting

A 1-day point-prevalence survey was performed annually between June and December from 2010 to 2017 in the Second Affiliated Hospital of Anhui Medical University which was opened in 2008. It is a tertiary care hospital with 2031 beds and had 70 968 patient admissions in 2017. It was accredited as an Academic Medical Centre Hospital by Joint Commission International (JCI) in January 2017. Clinical wards in this study were divided into the following eight groups: intensive care unit (ICU), departments of haematology, paediatrics and neonatology, oncology, internal medicine, surgery, gynaecology and obstetrics and others (consisting of dermatology, radiotherapy, invasive technology, rehabilitation and traditional Chinese medicine).

### Participants and HAI definitions

All inpatients including those who were discharged or had died in the hospital on the days of surveys were eligible for enrolment; outpatients and newly admitted patients were excluded from the study. HAIs were defined according to Diagnostic Criteria for Nosocomial Infection issued by the original Ministry of Health of the People's Republic of China (MHPRC) [14], adapted from the Centre for Disease Control and Prevention (CDC) [15]. Differences between MHPRC definitions and CDC definitions are shown in Table S1. Despite some differences between these criteria, the major infection types are broadly similar except for the category of lower respiratory tract infections (LRTIs) which by MHPRC includes the CDC categories of ‘pneumonias’ and ‘LRTIs other than pneumonias’ [16]. Likewise, patients aged <12 months are classified as a separate group by CDC definitions, but are included in the overall population by MHPRC, except for cardiovascular and central nervous system infections. In this study, HAIs were defined as being acquired more than 48 h after admission, but also included those acquired within 48 h of admission if there was a clear incubation period according to the definitions. Infections were classified as upper or lower respiratory tract infection (URTI/LRTI), gastrointestinal infection (GI), urinary tract infection (UTI), surgical site infection (SSI), skin and soft tissue infection (SSTI), bloodstream infection (BSI) and ‘others’.

### Training and data collection

A team in the Department of Hospital Infection Prevention and Control (HIPC) consisting of a microbiologist, epidemiologist, nurse, clinician and public-health physician took charge of survey design, staff training and data collection; the latter was performed by the team and HPIC part-time duty staff in each ward trained in the importance of data completeness and accuracy. Standardised survey questionnaire forms were completed by reviewing medical records and laboratory reports and visiting patients. Questionnaires were completed manually from 2010 to 2012 and subsequently through the hospital electronic information system from 2013 to 2017. The key variables of the survey questionnaire are shown in Table S2. Primary information collected included demographic and clinical data, HAI status, infection sites, microbiological cultures and antimicrobial use for all participants. Data completeness was checked by senior HIPC staff.

### Statistical analysis

The point-prevalence data were presented as the number of patients with at least one HAI or receiving at least one antimicrobial agent per 100 observed patients. The percentage frequencies of overall and specific HAIs were calculated according to the total number of patients and separately for individual clinical departments. Trends of HAIs and antimicrobial use over years were tested using the Mantel–Haenszel trend chi-square (*χ*^2^) test, and differences between the annual surveys were compared by the *χ*^2^ test. Statistical analyses were conducted by using the Statistical Package for the Social Sciences for Windows Version 13.0 (SPSS Inc., Chicago, IL, USA). All tests were two-tailed and *P* values <0.05 were considered as statistically significant.

## Results

Table 1 shows a total of 12 505 patients in the eight annual surveys from 2010 to 2017, in 2-year groups. Of these, 6904 (55.20%) were male and ages of all patients ranged from 2 days to 99 years. There were 600 HAIs identified in 533 patients with an overall prevalence of 4.26% (range: 3.03%–5.04%) and a frequency of 4.80% (range: 3.28%–5.64%). There was no evidence for an increasing or decreasing trend in prevalence over the study period (trend *χ*^2^ = 2.15, *P* = 0.143; Figure S1). However, a significant difference in HAI prevalence was evident between the surveys (*χ*^2^ = 21.14, *P* < 0.001). LRTIs were the most frequent (241 cases; 40.17% (relative proportions)), followed by URTIs (134 cases; 22.33%). Other infection sites each accounted for <10% of cases with GIs accounting for 58 cases (9.67%), UTIs for 38 cases (6.33%), SSTIs for 34 cases (5.67%), BSIs for 32 cases (5.33%) and SSIs for 20 cases (3.33%); with ‘other’ types identified in 7.17%. Notably, the relative proportion of BSIs significantly increased over the study period from zero to 5.96% (trend *χ*^2^ = 6.94, *P* = 0.008) while SSIs declined markedly from 7.69% to 0.92% (trend *χ*^2^ = 13.21, *P* < 0.001). ICU had the highest frequency of HAIs (24.36%) and some departments such as haematology (18.33%), paediatrics and neonatology (8.26%) and oncology (6.12%) had higher frequencies than the overall mean HAI frequency (Table 2).

**Table 1.** Prevalence and distribution of HAIs in a university hospital in Anhui, China, 2010–2017

Variables	2010–2011 (*N* = 2166)		2012–2013 (*N* = 3166)		2014–2015 (*N* = 3307)		2016–2017 (*N* = 3866)		Total (*N* = 12 505)	
	*n*	%	*n*	%	*n*	%	*n*	%	*n*	%
Prevalence of patients with HAI	109	5.03	96	3.03	133	4.02	195	5.04	533	4.26
Frequency of HAIs	117	5.40	104	3.28	161	4.87	218	5.64	600	4.80
Relative proportion of infection sites										
LRTIs	46	39.32	37	35.57	65	40.37	93	42.66	241	40.17
URTIs	24	20.51	23	22.12	34	21.12	53	24.31	134	22.33
GIs	16	13.68	7	6.73	15	9.32	20	9.17	58	9.67
UTIs	9	7.69	9	8.65	11	6.83	9	4.13	38	6.33
SSTIs	6	5.13	4	3.85	7	4.35	17	7.80	34	5.67
BSIs	0	0.00	3	2.88	16	9.94	13	5.96	32	5.33
SSIs	9	7.69	6	5.77	3	1.86	2	0.92	20	3.33
Others	7	5.98	15	14.42	10	6.21	11	5.05	43	7.17

**Table 2.** Distribution of HAIs in clinical wards in a university hospital in Anhui, China, 2010–2017

Specialty of ward	2010–2011			2012–2013			2014–2015			2016–2017			Total		
	*N*	*n*	%	*N*	*n*	%	*N*	*n*	%	*N*	*n*	%	*N*	*n*	%
ICU	15	3	20.00	47	12	25.53	39	10	25.64	55	13	23.64	156	38	24.36
Haematology	65	9	13.85	142	24	16.90	169	35	20.71	186	35	18.82	562	103	18.33
Paediatrics and neonatology	120	12	10.00	193	6	3.11	245	21	8.57	277	30	10.83	835	69	8.26
Oncology	286	25	8.74	444	10	2.25	488	25	5.12	497	45	9.05	1715	105	6.12
Internal medicine	685	31	4.53	1030	21	2.04	1034	42	4.06	1263	53	4.20	4012	147	3.66
Surgery	721	37	5.13	991	31	3.13	1011	21	2.08	1183	36	3.04	3906	125	3.20
Gynaecology and obstetrics	164	0	0.00	188	0	0.00	189	2	1.06	196	1	0.51	737	3	0.41
Others	110	0	0.0	131	0	0.00	132	5	3.79	209	5	2.39	582	10	1.72

Positive cultures of pathogens were obtained from 174 (29.0%) of HAIs. Gram-negative species predominated (64.37%), with *Escherichia coli* (16.67%), *Pseudomonas aeruginosa* (12.64%) and *Klebsiella pneumoniae* (11.49%) being the most common (Table 3). Gram-positive species collectively accounted for 18.39% with *Staphylococcus aureus* (7.47%) being the most frequent. *Candida albicans* (6.32%) was the most common yeast species identified.

**Table 3.** Distribution of HAIs pathogens in a university hospital in Anhui, China, 2010–2017

	2010–2011		2012–2013		2014–2015		2016–2017		Total	
	*n*	%	*n*	%	*n*	%	*n*	%	*n*	%
Gram-negative bacteria	32	62.75	30	83.33	19	57.58	31	57.41	112	64.37
* E. coli*	9	17.65	7	19.44	5	15.15	8	14.81	29	16.67
* P. aeruginosa*	6	11.76	7	19.44	4	12.12	5	9.26	22	12.64
* K. pneumoniae*	4	7.84	5	13.89	5	15.15	6	11.11	20	11.49
* Acinetobacter baumannii*	3	5.88	2	5.56	1	3.03	5	9.26	11	6.32
* Enterobacter cloacae*	5	9.80	3	8.33	1	3.03	–	–	9	5.17
* P. maltophilia*	2	3.92	3	8.33	–	–	–	–	5	2.87
* Citrobacter freundii*	1	1.96	–	–	–	–	–	–	1	0.57
* Morganella morganii*	1	1.96	–	–	–	–	–	–	1	0.57
* Burkholderia cepacia*	1	1.96	–	–	–	–	–	–	1	0.57
* K. ozaenae*	–	–	1	2.78	–	–	–	–	1	0.57
* Hafnia alvei*	–	–	1	2.78	–	–	–	–	1	0.57
Other Gram-negative bacteria	–	–	1	2.78	3	9.09	7	12.96	11	6.32
Gram-positive bacteria	9	17.65	3	8.33	9	27.27	11	20.37	32	18.39
*S. aureus*	3	5.88	1	2.78	4	12.12	5	9.26	13	7.47
* Enterococcus* spp.	3	5.88	2	5.56	1	3.03	2	3.70	8	4.60
* *Other *Staphylococcus* spp.	3	5.88	–	–	1	3.03	1	1.85	5	2.87
* *Other Gram-positive bacteria	–	–	–	–	3	9.09	3	5.56	6	3.45
Fungus	10	19.61	3	8.33	2	6.06	6	11.11	21	12.07
*C. albicans*	9	17.65	1	2.78	1	3.03	–	–	11	6.32
Other *Candida* spp.	1	1.96	2	5.56	1	3.03	–	–	4	2.30
Other fungi	–	–	–	–	–	–	6	11.11	6	3.45
Others	–	–	–	–	3	9.09	6	11.11	9	5.17
Total	51	100.00	36	100.00	33	100.00	54	100.00	174	100.00

In total, 5519 patients received one or more antimicrobial agents at the time of surveys, giving an overall prevalence of 44.13% which declined significantly over the study period from 53.60% to 40.95% (trend *χ*^2^ = 91.59, *P* < 0.001) (Table 4). Among patients who received antimicrobial agents, 61.08% were classed as for treatment, 24.81% for prophylaxis and the remainder (14.11%) for both purposes. Meanwhile, the relative proportion of therapeutic use of antibiotics also markedly increased from 43.50% to 63.93% over the years (trend *χ*^2^ = 83.83, *P* < 0.001). A single antibiotic was prescribed for 4069 patients (73.73%), two for 1271 patients (23.03%) and three or more for 179 patients (3.24%). Likewise, the relative proportion of monotherapy increased significantly from 65.55% to 72.33% (trend *χ*^2^ = 87.06, *P* < 0.001).

**Table 4.** Antimicrobial use in a university hospital in Anhui, China, 2010–2017

Variables	2010–2011 (*N* = 2166)		2012–2013 (*N* = 3166)		2014–2015 (*N* = 3307)		2016–2017 (*N* = 3866)		Total (*N* = 12 505)	
	*n*	%	*n*	%	*n*	%	*n*	%	*n*	%
Patients receiving antimicrobial agent	1161	53.60	1439	45.45	1336	40.40	1583	40.95	5519	44.13
Purpose of antimicrobial use										
Treatment	505	43.50	964	66.99	890	66.62	1012	63.93	3371	61.08
Prophylaxis	522	44.96	126	8.76	301	22.53	420	26.53	1369	24.81
Treatment and Prophylaxis	134	11.54	349	24.25	145	10.85	151	9.54	779	14.11
Therapy										
Mono therapy	761	65.55	1196	83.11	967	72.38	1145	72.33	4069	73.73
Dual therapy	370	31.87	185	12.86	337	25.22	379	23.94	1271	23.03
Triple (or multi-drug) therapy	30	2.58	58	4.03	32	2.40	59	3.73	179	3.24

## Discussion

This study is one of the longest duration prevalence surveys of trends and characteristics of HAIs, and antimicrobial use over time in China. Our results showed an overall 4.26% prevalence of HAI among 12 505 inpatients over an 8-year period. This rate was slightly higher than 3.9% found in 13 tertiary care hospitals in Hubei, China [16] and 3.7% in 54 tertiary care hospitals in different parts of China [17] and 4.0% in 183 hospitals in the USA [3], but was lower than previous surveys from other countries (4.6%–11.9%) [7, 18–21]. However, it is difficult to draw firm conclusions on the relative prevalence ranking of HAI through comparison within and between countries due to the confounding effect of differences in surveillance methods, case definitions and hospitals’ characteristics [16]. Although no significant increasing or decreasing trend in HAI prevalence was detected, a substantially lower prevalence (3.03%) was notable in 2012–2013. This was possibly due to the benefit of implementing multiple infection control measures including an improvement of hand hygiene facilities in clinical areas, the establishment of electronic surveillance system of infections, and effects of rewards and punishments in infection prevention and control in our hospital during that period. However, the greatest challenge was the gradually increasing number of inpatients, and so the HIPC department needed to prioritise measures to block the transmission of bacteria through optimal hand hygiene by staff, efficient environmental cleaning and disinfection and contact isolation of infected patients.

In line with previous studies [21–25], the ICU had the highest frequency of HAIs than other departments, most likely due to disease severity and widespread use of invasive procedures thus increasing the susceptibility of patients to infection. Of note, the distribution of most HAI types was quite different from previous surveys from China, ECDC and USA [3, 17, 21] as LRTIs and URTIs accounted for approximately two-thirds of HAIs in this study. Given that the MHPRC definition of LRTIs includes the CDC categories of ‘pneumonias’ and ‘LRTIs’, the combined relative proportion of such categories in studies from ECDC and USA were compared with findings in this study. We found that the relative proportion of LRTIs (40.2%) was higher than that from ECDC (25.7%) and USA (25.8%) [3, 21]. Likewise, our rate for GIs (9.7%) was markedly lower than that reported from the USA (17.1%) [3], but higher than ECDC (7.8%) and China (2.8%) [17, 21]. We also recorded a much lower prevalence of SSIs and UTIs than from all the above studies [3, 17, 21]. The significant increase in BSIs over the years is also noteworthy although appearing to decline in the most recent survey. Such data on changes in the frequency of infection sites over time should serve to inform the value of preferential and precise interventions to combat transmissions in the healthcare setting.

Only 29.0% of clinically defined HAIs yielded specific pathogens which was lower than previous reports [3, 17, 21]. This may have been due to the fact that some culture results were not available on the survey day, and emphasises the need to improve rapid microbiological confirmation of infection to avoid the potential misdiagnosis of HAIs. Overall, Gram-negative bacteria were isolated most frequently, which was in accordance with other studies’ results from China, ECDC and USA [3, 17, 21] with *E. coli* (16.7%) being predominant and similar to the results from ECDC (15.2%), but substantially higher than other reports from China (6.6%) and USA (9.3%) [17, 21]. The proportion of *S. aureus* (7.5%) found here also mirrors the study from China (6.6%) [17], but was notably lower than the USA (10.7%) and ECDC (12.1%) studies [3, 21].

The overall prevalence of patients receiving antimicrobial agents was 44.13%. It is noteworthy that the prevalence gradually dropped over the study period from 53.60% to 40.95%, which was most likely due to the impact of new legislation following a special campaign to promote the rational use of antibiotics implemented by the MHPRC throughout the country in 2011 [26]. The new management policies established a national antibiotic stewardship system, antibiotic formulary restriction in hospitals, setting up a task force to monitor usage and underpinned by legal liability [27]. Reassuringly, the proportion of therapeutic use of antibiotics (61.1%) compared favourably with studies from China (57.1%) and ECDC (66.4%) [17, 21]. The proportion of prophylactic use also dropped markedly and the use of single agents increased over the years, and reflects the success of measures to reduce antimicrobial use. Nevertheless, this study shows that a high number of patients continue to receive potentially inappropriate antimicrobials, and suggests that further measures to curb antimicrobial abuse and enhance their rational use are still required.

Some limitations in the current study are acknowledged. First, the rate of microbiological confirmation of HAIs was lower than that from similar studies [3, 21], which might have contributed to possible misdiagnosis of infections. Second, we were unable to further investigate the appropriateness of antimicrobial use owing to a lack of data on the antimicrobial susceptibilities of the isolated pathogens and the specific antibiotics used for treatment of patients with HAI.

In conclusion, our study suggests that the prevalence of patients with HAIs in our hospital was consistent, if slightly higher, than previous studies from China and lower than most other countries. More attention should be given to the surveillance and prevention as the highest risk of acquiring HAIs appears to be linked to clinical departments such as intensive care, and site of infection, notably the lower respiratory tract. Meanwhile, more efforts should be made to further enhance rational antimicrobial use.

## Supplementary material

For supplementary material accompanying this paper visit https://doi.org/10.1017/S0950268818002856. click here to view supplementary material
