# Supplementary material for: An 8-year point-prevalence surveillance of healthcare-associated infections and antimicrobial use in a tertiary care teaching hospital in China
Source: Epidemiol Infect. 2018 Oct 25;147:e31. doi: 10.1017/S0950268818002856 (PMC6518586; doi:10.1017/S0950268818002856)

***Epidemiology and Infection***

Title: An 8-year point-prevalence surveillance of healthcare associated infections and antimicrobial use in a tertiary care teaching hospital in China

Authors: Yi-Le Wu, Xi-Yao Yang*, Meng-Shu Pan, Ruo-Jie Li, Xiao-Qian Hu, Jing-Jing Zhang, Li-Qi Yang

Supplementary Material:

**Supplementary Table S1 Comparison of differences in major and specific types of health care–associated infections issued by Ministry of Health of the People’s Republic of China (MHPRC) and USA Centre for Disease Control and Prevention (CDC)**

| Items | MHPRC definition | CDC definition |
| --- | --- | --- |
| Major and specific types of infection | - Urinary tract infection - Not specially divided | - Urinary tract infection - Symptomatic urinary tract infection - Asymptomatic bacteriuria - Other infections of the urinary tract |
|  | - Surgical site infection (SSI) - Superficial incisional SSI - Deep incisional primary SSI - Organ/space SSI | - SSI - Superficial incisional primary SSI - Superficial incisional secondary SSI - Deep incisional primary SSI - Organ/space SSI |
|  | - Bloodstream infection - Catheter-related infection - Clinical sepsis | - Bloodstream infection - Laboratory-confirmed bloodstream infection - Clinical sepsis |
|  | - Respiratory system infection - Upper respiratory tract infection - Lower respiratory tract infection (Pneumonia included) - Pleural infection | - Pneumonia - Clinically defined pneumonia - Pneumonia with specific laboratory findings |
|  |  | - Lower respiratory tract infection, other than pneumonia - Bronchitis, tracheobronchitis, tracheitis, without evidence of pneumonia - Other infections of the lower respiratory tract |
|  | - Bone and joint infection - Osteomyelitis - Joint or bursa - Disc space | - Bone and joint infection - Osteomyelitis - Joint or bursa - Disc space |
|  | - Central nervous system infection - Intracranial infection - Meningitis or ventriculitis - Spinal abscess without meningitis | - Central nervous system infection - Intracranial infection - Meningitis or ventriculitis - Spinal abscess without meningitis |
|  | - Cardiovascular system infection - Endocarditis - Myocarditis or pericarditis | - Cardiovascular system infection - Arterial or venous infection - Endocarditis - Myocarditis or pericarditis - Mediastinitis |
|  | - Eye, ear, nose, throat, or mouth infection | - Eye, ear, nose, throat, or mouth infection - Conjunctivitis - Eye, other than conjunctivitis - Ear, mastoid - Oral cavity (mouth, tongue, or gums) - Sinusitis - Upper respiratory tract, pharyngitis, laryngitis, epiglottitis |
|  | - Gastrointestinal system infection - Gastroenteritis - Gastrointestinal tract - Hepatitis - Intraabdominal, not specified elsewhere - Ascitic fluid infection - Antibiotic associated diarrhea | - Gastrointestinal system infection - Gastroenteritis - Gastrointestinal tract - Hepatitis - Intraabdominal, not specified elsewhere - Necrotizing enterocolitis |
|  | - Reproductive tract infection - Endometritis - Episiotomy - Vaginal cuff - Pelvic inflammation - Other infections of the male or female reproductive tract | - Reproductive tract infection - Endometritis - Episiotomy - Vaginal cuff - Other infections of the male or female reproductive tract |
|  | - Skin and soft tissue infection - Skin - Soft tissue - Decubitus ulcer - Burn - Breast abscess or mastitis - Omphalitis - Newborn pustulosis | - Skin and soft tissue infection - Skin - Soft tissue - Decubitus ulcer - Burn - Breast abscess or mastitis - Omphalitis - Pustulosis - Newborn circumcision |
|  | - Systemic Infection | - Systemic Infection - Disseminated infection |
| Infection for patient ≤12 months of age | Not specially mentioned excepted for Cardiovascular system infection and central nervous system infection | Specially defined in most infections |

**References**

1. Ministry of Health of the People’s Republic of China. Standard for nosocomial infection surveillance. *Chinese Journal of Nosocomiology* 2009;19:1313-1314.
2. Garner JS, Jarvis WR, Emori TG, et al. CDC definitions for nosocomial infections, 1988. *American Journal of Infection Control* 1988;16:128-140.

**Supplementary Table S2** Case registration questionnaire of healthcare associated infections commonly used in a university hospital in Anhui, China, 2010-2017

| **Case Registration Questionnaire of Healthcare Associated Infections** |
| --- |
| Data collectors Date (day/mouth/year):　 / / |
| 1. Demographic and clinical data   1.1 Name: _  1.2 Gender:  Male □ Female □  1.3 Age: _  1.4 Medical record No.:  1.5 Clinical departments:  1.6 Bed number:  1.7 Number of admission: _  1.8 Admission date (day/mouth/year):　 / /  1.9 Admission diagnosis:  a 　 b  c 　 d   - 1. Surgical operation:   Yes □ No □   - 1. Operation type: |
| 1. **The status of healthcare associated infections**   2.1 Healthcare associated infections:  Yes □ No □  2.2 Infection site:  a  b  c  2.3 Pathogen:  a  b  c |
| 1. **The status of antimicrobial use**   3.1 Antimicrobial use:  Yes □ No □  3.2 Purpose of antimicrobial use:  Treatment □ Prophylaxis □ Treatment and Prophylaxis □  3.3 Therapy:  Mono therapy □ Dual therapy □ Triple (or multi-drug) therapy □ |

**Supplementary Figure S1** Trends in prevalence of patients with HAI and [frequency](../../../../C:%5CProgram%20Files%20(x86)%5CYoudao%5CDict%5C7.0.1.0227%5Cresultui%5Cdict%5C%3Fkeyword=frequency) of HAIs in a university hospital in Anhui, China, 2010-2017


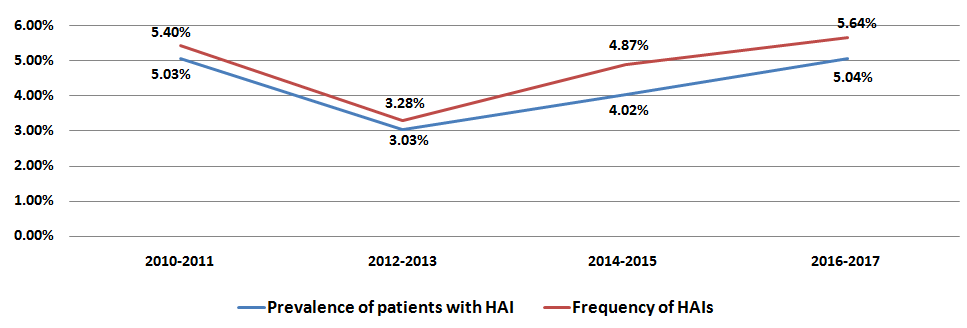

Supplement: Supplementary file 1 [file S0950268818002856sup001.doc]
